# Scorecard metrics for assessing the extent of integration of community health worker programmes into national health systems

**DOI:** 10.4102/phcfm.v13i1.2691

**Published:** 2021-11-26

**Authors:** Lucia M. Mupara, John J.O. Mogaka, William R. Brieger, Joyce M. Tsoka-Gwegweni

**Affiliations:** 1 Department of Public Health Medicine, College of Health Sciences, University of KwaZulu-Natal, Durban, South Africa; 2 Department of International Health, The Johns Hopkins Bloomberg School of Public Health, Johns Hopkins University, Baltimore, United States of America; 3 Faculty of Health Sciences, University of the Free State, Bloemfontein, South Africa

**Keywords:** CHW programmes, integration conceptual framework, health system measurement, integration metrics, sub-Saharan Africa

## Abstract

**Background:**

The effectiveness of community health workers (CHWs) in delivering community-based preventive services is often curtailed by inadequate or complete lack of integration of the CHW programmes into national health systems. Although literature has defined the context and guidelines for integrating CHW programmes into health systems, indicators to quantitatively assess the extent of integration are inadequately addressed.

**Aim:**

This article presents an integration scale – CHW Programme Integration Scorecard Metrics (CHWP-ISM) – for measuring the extent of CHW programme integration into national health systems.

**Setting:**

Literature review and policy documents were focused on sub-Saharan Africa, while interview participants were drawn from six African countries.

**Methods:**

A deductive–inductive approach to item and measurement scale development was employed. Information obtained from a combination of diverse literature sources, subject matter expert (SME) interviews and documentary abstraction from publicly available policy documents advised item generation for the proposed CHWP-ISM. The study qualitatively captured the sectoral CHW integration, thematically analysed the data and culminated in the quantitative integration metrics.

**Results:**

Analysis of the responses from six SMEs and abstraction from policy documents resulted in the compilation of metrics with a total of 100 indicators for the CHWP-ISM scale that could be used to assess the level of CHW programmes integration into national health systems. The indicators were categorised along the six World Health Organization’s (WHO) health systems building blocks. Subject matter expert responses corresponded well with abstracted results from the 18 country CHW programmes, indicating content validity.

**Conclusion:**

The proposed scorecard metrics can be used to quantitatively rate the extent of CHW programmes integration into health systems, in an attempt to strengthen health systems to improve health outcomes.

## Introduction

Community health workers (CHWs) possess a unique public health advantage that enables them to be more effective as compared with other healthcare cadres in the delivery of community-based health services. Some of the factors that enable CHWs to effectively deliver community-based health services are as follows: (1) they mostly belong to and operate within their local communities^1,2^, (2) they are trusted by local community members,^2,3^ especially where communities take part in CHWs selection themselves and (3) their cultural competency,^1,4^ proximity and availability in communities even beyond official working hours for local health facilities.^1,2^ Evidence highlighting CHWs’ potential and effectiveness in strengthening health systems to improve health outcomes by making services more accessible and acceptable has been steadily mounting.^5,6,7,8,9^

However, research also points out that the proved potency of CHWs in improving health outcomes is being curtailed by the lack of integration of CHW programmes into national health systems.^5,6,10,11,12,13,14^ Nevertheless, it has been reported that high-performing CHW subsystems are dependent on deep integration into health systems^5,15^ because they enjoy support from other health system actors. Integration of a CHW programme into formal healthcare systems has thus been pointed out as a critical component that is requisite to the design and implementation of effectual and maintainable CHW programmes.^16,17,18,19,20^ As a result, CHW programme integration has become a critical topic of international deliberations and concern to the global public health community, as evidenced by current World Health Organization’s (WHO) recommendations to member states to integrate CHW programmes into national health systems.^2,21,22,23^

Recent literature has defined the context, mechanisms and guidelines for integrating CHWs programmes into mainstream health systems,^5,6,21,22,23,24,25,26,27,28,29,30^ but not many studies have quantitatively assessed the extent of CHW programmes integration into respective health systems in sub-Saharan Africa (SSA). This study aimed at identifying the integration metrics for assessing the extent of CHW programmes integration into national health systems in SSA. The study also undertook content validity for the identified scale items. This study adopted the definition of integration by Atun et al.,^31^ which states ‘the extent and nature of adoption and eventual assimilation of CHW programme into each of the critical functions/building blocks of a health system’.

## Methodology

The study employed a deductive–inductive approach to item and measurement scale development. Information was obtained from a combination of literature reviews, interviews with field experts and documentary abstractions from publicly available policy documents of 18 sub-Saharan African countries. The study formulated a conceptual framework that guided the integration metrics item identification and development.

### Participants

The study used two types of sources of information: the subject matter experts (SMEs) and documents (the country’s CHW programme policies). Purposive and snowballing sampling techniques were used to identify six SMEs for the purpose of item generation through in-depth interviews between the months of July and November 2018. Expertise of SMEs was judged based on their involvement in CHWs’ work as evidenced by the publication history in peer-reviewed journals and participation in key CHW forums. Six SMEs agreed to participate in the interview survey.

Documents used for this study were conveniently sampled from Community Health Systems (CHS) Catalogue database (https://www.advancingpartners.org/resources/chsc). They comprised of country-specific CHS Catalogue produced by the United States Agency for International Development’s (USAID’s) Advancing Partners and Communities (APC) project (APC 2017). The CHS Catalogue is an online CHW resource pool that contains information on community health programmes, CHWs and CHW interventions. We focused on CHS Catalogues because they contained summaries of country-specific CHW programme policies, strategies, guidelines, plans and reports. The CHS Catalogue is based on the WHO’s health systems framework, which is the premise upon which this study’s integration variables are built. Further inclusion criteria comprised of catalogues that focused specifically on sub-Saharan African countries, community health programmes, CHWs and CHW interventions at policy level.

### Procedure

The study was conducted in a three-step procedure. The first step was literature and documentation review of CHW programmes that led to the identification and definition of the construct under study, which is integration of CHW programmes into national health systems. It was also used to deductively arrive at scale item generation. Item and scale development procedure employed the conceptual framework, which is described in the next section.

The second step involved SME interviews. Subject matter experts who were locally based were interviewed in person, whereas those who were out of the country were mainly interviewed telephonically (Skype), using unstructured but themed interviews. An interview guide based on the six integration variables was used to elicit respondents’ views rather than open-ended questioning. Before each interview, SMEs were briefed and an operational definition of integration was provided to guide their understanding of the study concepts. Subject matter experts were asked to respond to how each of the a priori identified CHW integration variables would assess the extent of CHW programme integration into national health systems. Their responses were qualitatively recorded and thematically presented in Table 2, which is part of generated items.

The third step involved scale item content validation. This was conducted through documentary abstraction from publicly available CHS policy documents. We started by establishing if the country has policy documents that articulate the inclusion of CHW work. Thereafter, attention was directed at abstraction of evidence of integration for each WHO building block from the available policy documents. A web-based form was used to integrate the data abstracted from the policy documents scored as ‘YES’ or ‘NO’ to indicate the presence or absence of the evidence of integration at policy level. This was to facilitate the collection of data with minimum cost, ensure upsurge in response rate and allow for instant feedback, as advised in Refs.^32,33^ The determination of ‘YES’ or ‘NO’ was done by the researchers based on pre-agreed criteria for assessing the evidence of integration. Two researchers abstracted the data from the policy documents. The first four policy documents were abstracted in parallel to find the level of agreement between the two based on the data abstraction form. Disagreement was resolved by a moderator. The policy data abstraction were carried out not only to assess the extent to which questions imitate the construct under study and that responses produce valid measurement, but also to ensure availability of enough data for scale development and validation as advised by Boateng et al.^34^ The results are shown in Appendix 1. Data amassed through the given methods were analysed thematically to identify key integration themes and corresponding integration variables, parameters and indicators.

In summary, through existing literature on CHW programmes related to possible indicators^5,21,22,23,24,26,27,35,36,37,38^ of the integration construct,^39,40^ interviews of the SMEs^41^ were used to deductively^42^ and inductively^39,42^ identify items for the integration measurement scale. Two methods were employed to define and identify the items that assess the integration of CHW programmes into national health systems at this stage to facilitate a balance of both the theoretical basis and manifest forms^34^ that give a broader and more comprehensive^43,44^ scale of measurement. Content validity was performed through documentary abstractions.

### The community health worker programme integration variable identification framework

**FIGURE 1:**
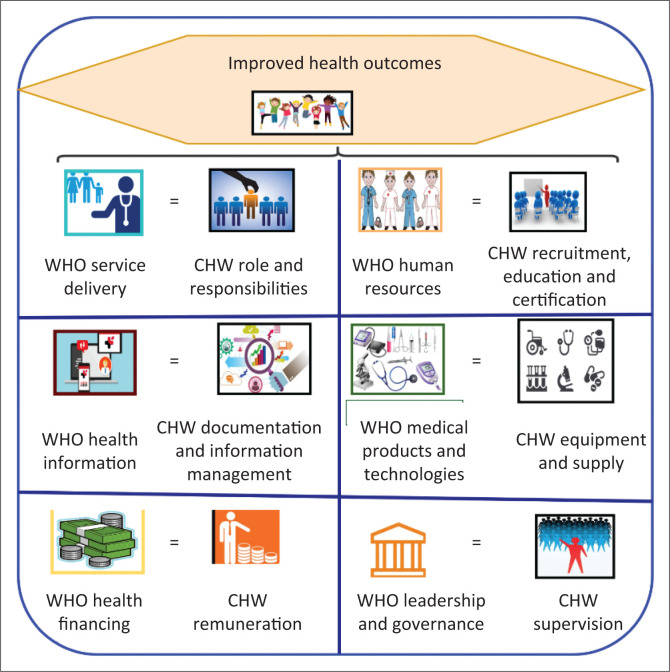
Community health worker programme components chosen for the study and how they correspond with the World Health Organization’s health systems building blocks.

The dimensions of the domain under study were determined a priori, as contained in Boateng et al.^34^ The integration of CHW programmes into health system was premised on the WHO’s Health Systems building blocks framework for health systems strengthening (HSS).^45^ Specific efforts of CHW programmes in strengthening health systems were set at improving the already existing blocks which are as follows: service delivery, human resources for health, information, medical products, financing and leadership and governance as stated in the WHO’s building blocks.^45^ The WHO’s building blocks framework has been criticised for failure to acknowledge the interconnectedness and interaction of the various health systems components, as well as customers and communities who are the critical core of health systems. However, the adoption of the framework for this study was premised on the fact that some of the concerns have since been addressed in a revised version of the framework.^46^ The study also employed some of Perry et al.’s interlinkages between the WHO’s health systems building blocks^45^ and CHW programmes,^27^ as explicated in the 2013 CHW Assessment and Improvement Matrix (AIM) best practice for CHW programme functionality.^26^ For the purpose of this study, the corresponding CHW programme components that seek to strengthen respective six critical functions of the health system to improve health outcomes were identified as CHW recruitment, education and certification (REC)^21,22,23^, CHWs’ roles and responsibilities, CHWs’ remuneration, CHWs’ supervision, CHWs’ information management and CHWs’ equipment and supplies^22,23,26,27,35^ for the development of the measurement metrics scorecard as illustrated in Figure 1.

Based on extant literature, the CHW programme components illustrated in Figure 1 were incorporated and used to build a more parsimonious yet robust CHW integration framework. In addition to the WHO’s health systems building blocks and their corresponding CHW programme components, this study used three more parameters by which the construct of integration was examined at a more refined resolution: the process of integration, the evidence of integration and status of integration. To satisfy the process of integration parameter, selected countries had to have policy documents that clearly articulated procedures for the inclusion of CHW work. Availability of government/Ministry of Health (MOH) policy-generated standardised guidelines on each of the CHW programme components that seek to improve corresponding WHO’s health systems building blocks provided evidence of integration.

**FIGURE 2:**
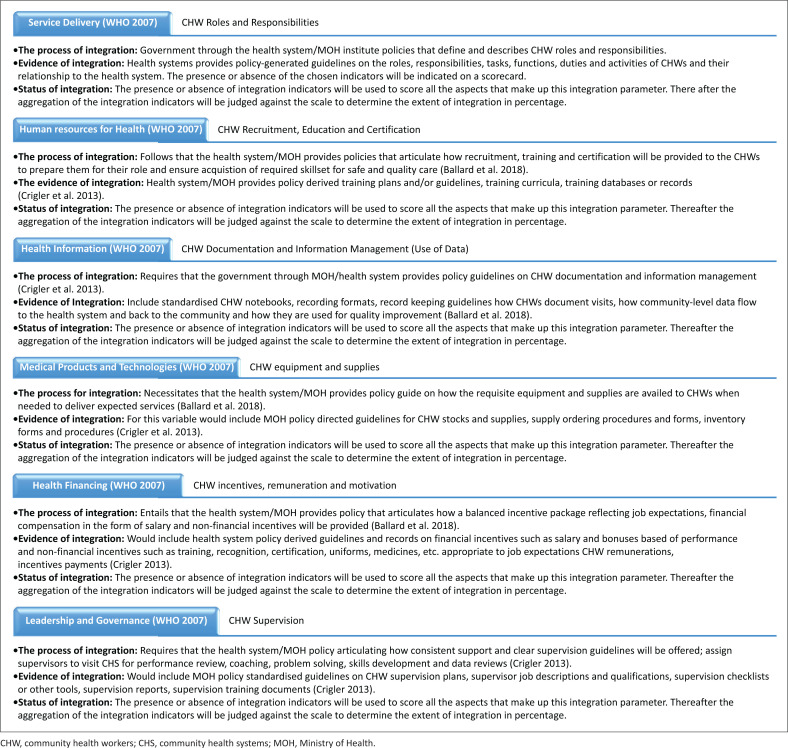
Framework for assessing the extent of community health worker programmes’ integration into health systems.

Lastly, the status of integration zoomed into the actual extent or degree of integration. This component is adopted from the study by Atun et al.,^47^ who proposed that integration status of health interventions (CHW programmes) into health systems can assume different forms. They could either be fully, partially or not integrated at all, in line with the respective building blocks of the health systems.^31,48,49^ Based on CHW integration components as illustrated in Figures 1 and 2, this study proposes a quantitative interpretation of CHW programmes integration. The presence or absence of integration indicators is used to score all the aspects that make up specific integration parameters. Thereafter, the aggregation of the integration indicators is judged against the scale to determine the extent of integration (expressed as a percentage). Therefore, drawing from existing literature,^26,27,35,37,45,47,50^ this study used an integration conceptual framework (Figure 2) within which an integration scorecard metrics was developed, whose core elements are:

- WHO’s health systems building block
- corresponding CHW programme component
- process of integration
- evidence of integration
- status of integration

### Ethical considerations

Ethical approval to conduct the research was granted by the Biomedical Research and Ethics Committee of the University of KwaZulu-Natal (BREC – BE066/17). Permission to carry out the research was obtained from the Health Research and Development Division of the University of KwaZulu-Natal.

## Results

### Demographic characteristics of human participants

All six SMEs who participated in this study had more than 15 years of working experience in primary healthcare (PHC) and CHW work at various designations. Moreover, of the six SMEs interviewed, one had both Medicine and Master of Public Health (MPH), another had MPH, whilst the rest had PhDs as their highest attained degree qualifications. Of the six participants, there were two males and four females. The youngest participant was aged 40 years, whilst the oldest was aged 75 years. The SMEs were from Botswana, Ethiopia, Kenya, Malawi, Nigeria and Zambia.

### Summary of subject matter expert responses

Table 1 presents thematically summarised SMEs’ interview responses. In the table, the six SMEs are represented by letters ‘A’ through ‘F’. The first column presents the prior identified CHW integration components. Subject matter experts responded to how each of the integration variables would assess the extent of CHW programme integration into national health systems. They were further asked to suggest suitable indicators that could be used to measure the extent of CHW programme integration into corresponding health systems building blocks. More generally, respondents suggested many integration indicators, which were well represented amongst the six critical functions of a health system, although the CHW supervision did not have as much indicators as did the other CHW programme components. Regarding the indicators of integration of CHW REC, all SMEs suggested the use of accredited and standardised training curricula for CHWs. Others suggested that integration indicators for this component were inclusion of recruitment processes, selection criteria, standardised training modalities and certification and provision of government sponsorship for training in policy documents.

**TABLE 1:** Subject matter experts’ responses.

CHW programme component/integration variable	Subject matter experts (SMEs)					
	A	B	C	D	E	F
CHW recruitment, education and certification	National accreditation of CHW training curricula Certification of CHWs Standardised CHW training	CHW training sponsorship Defined recruitment process Standardised training curriculum	National accreditation of CHW training curricula Standardise training modalities Recruitment process	Standardised training modalities Use of accredited CHW training curricula	Selection criteria and recruitment process Certification of CHWs Standardised CHW training	National accreditation of CHW training curricula Selection criteria specification
CHWs’ role and responsibilities	CHW services to be linked to the health system Signed contractual agreements between CHWs and employers	Defined scope of CHW services CHW responsibilities to be described in policy CHW services to be linked to the health system	Signed contractual agreements between CHWs and employers Defined scope of CHW services CHW services to be linked to the health system	CHWs’ roles articulated in policy CHW responsibilities to be described in policy	CHWs’ responsibilities to be described in policy Defined scope of CHW services CHW services to be linked to the health system	Signed contractual agreements between CHWs and employers Defined scope of CHW services CHW services to be linked to the health system
CHW remuneration (including financial and non-financial incentives)	Standardised financial incentives package Work aids, e.g., bicycles, motorbikes Preferential access to health	CHWs salaries Opportunity for career development Health insurance	Standardised non-financial incentives Work uniforms, e.g., T-shirts, name tags, badges, umbrella	Performance-based allowances Work aids such as cellphones, flashlights	Policy to indicate government funding for CHW incentives Work uniforms, e.g., boots, raincoats, backpacks	Social recognition by communities Formal recognition by other health workers Opportunity for professional development CHW salaries
CHW supervision	Mechanisms for supervision Health system to provide CHW supervision	Performance evaluation Coaching and mentorship	Strategies for supportive supervision Technical support	CHW performance evaluation	Supervision modalities Administrative support	Strategies for supportive supervision Supervisor-to-supervisee ratio
CHWs’ information management	CHWs’ supervisor role in data collection Community data integrated in national health information system	Data confidentiality issues Standardise data collection tools Use of community generated data to solve local problems	Mechanisms for data collection Mention of use of digital technologies Community data integrated in national health information system	Data security measures articulated in policy Use of community generated data to inform programmatic improvement	CHWs’ role in data collection Use of community generated data to inform programmatic improvement Data confidentiality	Consolidation of community and facility data CHWs’ supervisor role in data collection Community data integrated in national health information system
CHW equipment and supplies	Defined procedure followed for equipment and supply stocking Supplies quality check	Safe disposal of medical waste generated through community service Provision of job aids for CHWs	Specified processes for ordering for equipment and supplies Mechanisms for supplies forecasting	CHW supplies inventory Availing CHW job aids Mechanisms for supplies backup	Use of official procedures to obtain supplies Adequate commodity storage	CHWs’ equipment and supply provision mechanisms Registration of obtained supplies and equipment

CHW, community health worker.

With regard to CHWs’ roles and responsibilities component, five SMEs indicated the linkage of CHW services to the health systems as the key indicator of integration. The other suggested integration indicators under this CHW component were clear definition of CHWs’ roles, responsibilities and scope of work at policy level. In particular, CHW remuneration attracted responses that indicated that policy documents should include both financial and non-financial incentives that may be employed to motivate CHWs to stay on the job, as indicators of integration. For instance, some SMEs expressed that in addition to just salaries and wages, work-related incentives such as provision of work aids, equipment and uniforms by the health systems could be used as indicators of CHW integration into national health systems. Community health workers’ supervision had the most varied integration indicators from respondents ranging from the policy inclusion of supervision mechanisms and strategies, supervisor–supervisee ratios, provision of technical and administrative support, coaching and mentorship to CHWs’ performance evaluation.

Meanwhile, the CHW information management component had two most featured integration indicators amongst the SMEs: the integration of CHW generated data into national health information system and consolidation of community and facility data. The other suggested integration indicators for this component were the use of standardised data collection tools and the definition of the roles of both CHWs and their supervisors in data collection. In addition, three SMEs suggested the inclusion of data confidentiality and data security mechanisms in policy documents. Lastly, SMEs suggested varied indicators for CHW equipment and supplies component. Some of the suggestions were inclusion of mechanisms and procedures (processes) of CHW equipment and supply provisions in policy documents. In addition, they also suggested policy articulation on safe disposal of medical waste generated through community service. The rest of the integration indicators for all the six CHW programme components are summarised in Table 1.

### Summary of data abstractions for 18 countries

This section presents summarised characteristics of CHW programmes reviewed in terms of the region where the country is located in SSA, country name, CHW programme name and CHW cadre name. Out of a total of 18 CHW programmes under consideration, 8 were from West Africa, 5 from East Africa, 4 from Southern Africa, whilst there was 1 from Central Africa. The rest of CHW programme characteristics are in Table 2, whilst online Appendix 1 presents the data abstracted from policy documents and scored as YES or NO to indicate the presence or absence of evidence of integration at policy level. Appendix 1 presents the CHW Programme Integration Scorecard Metrics (CHWP-ISM) scorecard metrics in the form of thematically synthesised data on CHW programme components, integration parameters and integration indicators. Items for CHWP-ISM scale were generated from CHW programme components that map onto respective WHO’s health systems building blocks. The SMEs’ responses presented in Table 1 correspond with the abstraction results in online Appendix 1, showing content validity. Therefore, Appendix 1 presents a consensus arrived at through a synthesis of data obtained from three sources: literature review, SME interviews and document abstractions.

**TABLE 2:** Community health worker programme integration documentary abstraction results of the 18 sub-Saharan countries.

Region in SSA	Country	Name of CHW programme	Name of CHW cadre
Central Africa	DRC	Programmes across different health areas (e.g. reproductive health and nutrition) have community components.	One main cadre: relais communautaires (RECO)
East Africa	Rwanda	Community-based Nutrition Programme (CBNP); Integrated Community Case Management (iCCM) Programme; Community Maternal New-born Health; Community-based Provision of Family Planning; Non-communicable Diseases and HIV/AIDS	Agents de santé maternelle (ASM) Binomes
	Ethiopia	Health extension programme	Health extension workers (HEWs)
	Kenya	Programmes are implemented at the county level, and therefore differ	Community health extension worker (CHEW)
	Tanzania	(1) Community-based Health Programme (2) Mpango wa Maendeleo wa Afya ya Msingi (Primary Health Service Development Programme)	Community health worker (CHW)
	Uganda	CHEW; iCCM; RMNCH; national TB and Leprosy Control and HIV/AIDS Control programmes; Expanded Programme on Immunization.	Community health extension workers (CHEWs)
Southern Africa	Madagascar	Various programmes in specific health areas including nutrition, malaria and reproductive health	Agents communautaires (ACs)
	Malawi	National Health Surveillance Agent (HSA) Programme	Health surveillance agents (HSAs)
	Mozambique	APE Programme; various programmes in specific health areas including nutrition, malaria and reproductive health	Agentes polivalentes elementares (APEs)
	Zambia	Community Health Assistants Programme	Community health assistants (CHAs)
West Africa	Benin	National Community Health Support Programme (PASCom)	Relais communautaires (RCs)
	Ghana	Community-based Health Planning and Services (CHPS)	Community health officers (CHOs)
	Liberia	Community Health Assistants (CHAs) Programme	Community health assistants (CHAs)
	Mali	Various national health programmes in multiple health areas	Community health agents (ASC)
	Nigeria	Primary healthcare (PHC) system	Community health extension worker (CHEW) Community health officer (CHO) Junior community health extension worker (JCHEW)
	Senegal	Community Health Programme (PSSC); Bajenu Gox Programme, other programmes in health-specific areas (e.g. TB, malaria, HIV and AIDS)	Agents de santé communautaire (ASC) Bajenu gox Dispensateurs de santé domicile (DSDOM) Matrones Relais commun-autaires (relais)
	Sierra Leone	National CHW Programme	Community health workers (CHWs)
	South Sudan	Various community health programmes aligned under the PHC service delivery system	Community health workers (CHWs) Community midwives (CMWs) Home health promoters (HHPs) Maternal and child health workers (MCHWs) Traditional birth attendants (TBAs)

As indicated in online Appendix 1, most integration parameters scored variedly across the 18 countries. With regard to CHW REC, most countries that scored positively in all indicators save for government sponsorship for CHW training, which was not articulated in all the reviewed country policy documents. Another integration indicator that was missing in all reviewed countries is government policy requirement for CHW training providers’ accreditation by a national accreditation board and policy guidance on CHWs’ post-qualification academic and career progression pathways. For instance, Liberia’s policy that provides guidance on CHWs’ post-training accreditation should be carried out by the national accreditation board, whilst all other countries were silent on the matter.

The CHWs’ roles and responsibilities and the CHWs’ services linkage to health systems integration parameters were positively scored in the policy documents of all countries. However, Tanzania was the only country with evidence of all five indicators of the CHWs’ contractual agreements integration parameter. With regard to CHW services linkage to health system integration parameter, most countries fared very well except for description of transport system availed to get CHW-referred clients to the referral facility, which was missing in the policy documents and policy guidance on CHWs’ use of standardised referral tool for clients to take to the facility, which was provided by Nigeria, Tanzania and Zambia only.

The CHW remuneration component was fairly scored across all countries, as varied policy guidance was found on different integration indicators. Community health workers supervision parameter was generally poorly scored on most indicators in all countries, except for policy guidance on the need for community health work to be managed across all levels of the health system, which was found on all country policy documents. However, no policy guidance was found on appropriate supervisor-to-supervisee ratio in all countries reviewed. In addition, guidance on how CHW performance is managed and evaluated by supervisors was not found for all reviewed countries.

The CHWs’ information management component was highly scored by all countries with indicators such as training of CHW supervisors to verify and audit data for the quality stipulated in policy documents. The most poorly scored indicator for this parameter was policy documents’ prescription on the use of standardised data collection tools, which was found only in Senegal, Sierra Leone, Tanzania, Uganda and Zambia. The CHWs’ equipment and supplies component was sporadically scored with outlier indicators such as policy guidance on provision of necessary equipment, supplies and job aids to CHWs and list of commodities for selected interventions that CHWs offer according to the country’s policy guidelines found for all countries. However, other integration indicators were poorly scored. For instance, policy documents stressing the need for regular CHW supplies updates for quality check was stipulated for Mali and Mozambique only. Still on this component, some indicators were fairly scored, as evidenced by policy guidance on the safe disposal of medical waste generated through CHW service, which was articulated for 10 countries, whilst the remaining 8 countries were silent. The rest of the combined results for item generation and content validation from literature review and documentary abstraction, respectively, are summarised in online Appendix 1.

## Discussion

This study set out to identify integration metrics for assessing the extent of CHW programmes integration into national health system in SSA. The study also undertook content validity for the identified scale items. This section thematically discusses the proposed components of CHWP-ISM measurement scale components, as analysed using data from literature and documentation review, interviews with field experts and documentary abstractions. The framework used to guide identification of scale items comprised of HSS building blocks, corresponding CHW programme component, process, evidence and status of integration. The proposed items and content validity results in Appendix 1 and online Appendix 1 comprise of HSS building blocks, analogous CHW programme component, integration parameters and indicators.

### Community health worker recruitment, education and certification (human resources for health)

According to SMEs’ interviews, literature and documentary abstraction outcomes, as shown in Tables 1 and 2 and online Appendix 1, specific integration parameters for CHW REC include; the presence of selection criteria and recruitment process, full government sponsorship for CHW training, CHW training programme accreditation and CHW standardised training modalities in policy documents. Health workforce contributes to the performance of the health systems. This includes being available, competent, responsive and productive.^45^ In supporting this assertion, our results, just as other prior works on CHWs,^5,51,52,53^ point to training as one of the main means of assessing CHW integration with respect to REC.

This study found evidence of process of integration at policy level by examining policy articulation of how CHW recruitment/selection criteria were conducted and how pre-service training and certification were provided to the CHWs to prepare them for their roles and ensure acquisition of required skill set for safe and quality care. This resonates with other recommendations from research that CHW programmes must ensure that a core set of skills and information is provided to CHWs.^5^ The evidence of integration sought for under this integration variable was therefore health system policy-derived CHW recruitment processes and selection criteria, training plans and/or guidelines, training curricula, training databases and certification. This resulted in the 20 items identified that may be used to quantitatively indicate CHW programme integration under REC, as shown in Appendix 1.

### Community health workers’ roles and responsibilities (service delivery)

According to our findings, specific integration parameters that could be used to measure the extent of CHW programme integration into the service delivery building block include articulation of CHWs’ services linkages to the health systems, CHWs’ scope of service, CHWs’ roles and responsibilities and signing of contractual agreements between CHWs and their employers at policy level. These findings resonate with the earlier recommendations that national decisions on the services provided by the CHWs should apex into a minimum package of activities defined by the country policy grounded on the countries’ epidemiological and community priorities.^9^ This is because CHWs represent one of the channels for health service delivery through which preventive, curative or rehabilitative care^45^ could be delivered to populations at the home, the community, the workplace or in health facilities.^45^

Therefore, community health workers could be considered as an extension of the healthcare system that takes healthcare to the household and community levels.^9^ As such, the amalgam of services delivered by the CHWs should mirror an integrated set of community health interventions that feed obviously into the service delivery building block of the health system. The study found evidence of the process of integration for this variable by evaluating the policy articulation for defining and describing the scope of their work in terms of roles and responsibilities.^26^ Evidence of integration zoomed into policy-derived guidelines clarifying the CHWs’ roles, responsibilities, tasks, functions, duties and activities of CHWs and their relationship to the health system.^35^ This resulted in the 15 indicators identified that may be used to quantitatively indicate CHW programme integration under REC, as shown in Appendix 1.

### Community health workers’ remuneration (health financing)

According to SMEs’ interviews and document abstraction outcomes shown in Table 1, Appendix 1 and online Appendix 1 parameters for CHWs’ remuneration include; the articulation of information on government funding for CHWs’ incentives, CHWs’ contractual agreements and guidelines on standardised packages for both financial and non-financial incentives in the policy documents. Our findings resonate with recommendations from other reviews that remuneration and provision of incentives for CHWs are essential and should be included in the health financing plan of the health systems.^5,9,23^ In particular, the health system/MOH should provide policy that articulates how a balanced incentive package reflecting job expectations, financial compensation in the form of salary and non-financial incentives will be provided.^35^ This is also in sync with WHO’s recent strong recommendation to remunerate practicing CHWs for their work with a financial package commensurate with the job demands, complexity, number of hours, training and roles that they undertake.^21,23^

This recommendation could have come as a response to earlier research findings that indicated lack of befitting remuneration as one of the renowned factors that diminish motivation and quality of work amongst CHWs.^9^ This study found evidence of integration at policy level by examining policy articulation on guidelines and records on financial incentives such as salary and bonuses based on performance and non-financial incentives such as training, recognition, certification, uniforms and medicines appropriate to job expectations, CHWs’ remunerations, incentives and payments.^26^ This resulted in the 20 items identified that may be used to quantitatively indicate the extent to which CHWs’ remuneration is integrated with the health financing building block of the health system as shown in Appendix 1.

### Community health workers’ supervision (leadership and governance)

According to our findings, specific integration parameters that could be used to measure the extent of CHW programme integration into the leadership and governance building block include articulation of supervision mechanisms and modalities, supportive supervision strategies and CHWs’ performance evaluation by supervisors in policy documents. These findings harmonised with other recommendations that health system/MOH policy should describe how consistent support and clear supervision will be offered and assign supervisors to visit CHS for performance review, coaching, problem-solving, skills development and data reviews.^26^ This buttresses several valuations of existing CHW programmes that cited lack of proper CHW supervision as one of obstacles to delivery of quality community health services by CHWs.^5,38,51,52^

In response, direct and supportive supervision of CHWs by facility-based staff has been recommended as fundamental to strengthen integration of CHWs into health system.^9,21,23^ This study found evidence of integration at policy level by examining policy articulation on CHW supervision plans, supervisors’ job descriptions and qualifications, supervision checklists or other tools, supervision reports and supervision training documents.^35^ This elicited 20 items identified that may be used to quantitatively indicate the extent to which CHW supervision is integrated into the leadership and governance building block of the health system as shown in Appendix 1.

### Community health workers’ documentation and information management (health information)

According to the SMEs’ interviews and literature outcomes shown in Table 1 and Appendix 1, respectively, specific integration parameters that would indicate if CHWs’ information management is integrated in the health information critical function of the health system include policy articulation of mechanisms for data collection and definition of both CHWs and their supervisors in data collection. These findings resonate with earlier recommendations that governments through MOH/health system ought to provide policy guidelines on CHW documentation and information management,^26^ particularly pronouncing how CHW collected data streams to the health system and back to the community and the use of the data for improving quality of care^35^ at community level. This is important because health knowledge and information gathered from different data sources via sundry channels is needed for decision-making at all levels of the health systems.^49^ Community health workers are one of the channels through which important and hard-to-get health data can be mined from households.

The Earth Institute Technical Taskforce report submitted that the unique ability of CHWs to capture multifaceted and dynamic epidemiological and community swings at household level supplies critical information that advises health systems of their performance, especially with regard to local and national health priorities.^9^ In this study, evidence of integration sought for under this CHW programme component included policy articulation on standardised use of data collection tools such as CHW notebooks and recording formats. We also examined policy pronunciation of record-keeping guidelines on how CHWs document visits, how community-level data flow to the health system and back to the community and how it is used for service improvement.^26^ This culminated into 15 items that may be used to quantitatively indicate CHWs’ information management and its integration into health information, which is a critical function of the health system as shown in Appendix 1.

### Community health workers’ equipment and supplies (medical products and technologies)

Our findings from both literature review and SMEs’ interview show that integration parameters that could be used to specify if the CHW programme is integrated into the health systems’ medical products and technology’s critical function include mentioning of CHWs’ equipment and supply mechanisms, procedures and processes in the policy documents. The findings resound with other recommendations that the health system/MOH should provide policy on how CHWs avail the necessary equipment and supplies when needed to deliver expected services.^35^

Research has demonstrated how CHWs’ acceptability in the community is closely tied to commodity availability.^5^ It has been documented that if CHWs do not have a necessary supply to perform their duties, they lose credibility from the families they serve and will be rendered ineffective over the long term in improving child health outcomes.^9^ Community health workers should therefore be furnished with a sturdy supply of all commodities and material support essential for their daily service delivery. This study found evidence of integration at policy level by examining policy articulation on guidelines for CHWs’ stocks and supplies, supply ordering procedures and forms, inventory forms and procedures.^26^ This culminated into 15-scale measures identified that it may be used to quantitatively indicate CHWs’ equipment and supplies’ integration into health information critical function of the medical products and technologies as shown in Appendix 1.

In developing the scorecard, the study made the assumption that all the six health systems building blocks contribute equally to the improvement of health outcomes. Further research is needed to ascertain contributory weights of each critical function.

## Conclusion

The study aimed to identify scale items for a CHW integration scorecard metrics (CHWP-ISM) for assessing the degree of integration of CHW programmes into national health system in SSA. The proposed CHWP-ISM draws CHW integration variables that correspond with WHO’s framework for HSS. It views the seamless and deliberate blending of CHW programme integration variables with critical national health system building blocks as one way of strengthening health systems to improve child health outcomes. Its use of WHO’s six building blocks for health systems makes it applicable for evaluating the extent of integration of CHW programmes into the critical functions of the health system. This was intended to better the existing integration literature by juxtaposing CHW functions with the WHO’s health system building blocks and in articulating how the former can be positioned to strengthen the latter. In other words, the study sought to examine not only how the health system structurally maps onto all essential CHW facets (integration variables) but also how these CHW facets link back to the various building blocks of a health system. This was explored with particular emphasis on public policies and guidelines that guide CHW programmes through evidence of CHWs inclusion into health system planning, budgeting and provision of logistical support.^35^

This CHWP-ISM can be mainly used to quantitatively assess CHW programmes’ extent of integration at national health systems level. But it may also, to some extent, be used to assess extent of CHWs at other levels of the health system, for example, local, district or provincial, particularly to indicate the interaction between the respective components of the health intervention under study and its corresponding WHO building block of the health system. The institutions and organisations that could benefit from the use of these recommendations include policymakers and advisers, health systems, ministries of health, donors, non-governmental organisations, programme managers and implementers and public health practitioners to mention but a few.

It is hoped that the use of this CHWP-ISM to assess the integration of CHW programmes can identify gaps that once addressed could better strengthen health systems to improve health outcomes in sub-Saharan Africa. For example, the scorecard recommends the involvement of community members and structures in CHWs’ selection and recruitment. The scorecard can be used to identify a gap in this regard. Addressing this gap ensures that communities accept the CHWs and the health services that they offer. This enhances universal health coverage and achievement of health-related SDGs. However, the CHWP-ISM measurement scale needs to be subjected to statistical analysis, especially factor analysis to determine its dimensionality, construct validity and internal consistency in assessing the degree of integration of CHW programmes into national health systems.

## Study limitations

The present study focused on policy level evidence of integration. This has a limited bearing on the gap between policy and practice, and hence, further research is needed to measure the evidence of integration of CHW programmes at implementation level. In addition, the study used the data collected from SMEs who were available during the short data collection window only. This also has a limited bearing on the kind of integration insights that might have been gained were it to extend to time that other SMEs would be available.

## Appendix 1

**TABLE 1-A1:** Community Health Worker Programme Integration Scorecard Metrics (CHWP-ISM).

CHW programme component and health systems building block	Integration parameter	Integration indicators	YES	NO
1. CHWs’ recruitment, education and certification (Human resources for health)	1.1 CHW selection criteria and recruitment process	1.1.1 Policy documents stipulate entry level educational qualifications as part of selection criteria		
		1.1.2 Policy documents include community membership or residency as part of selection criteria		
		1.1.3 Policy guidance on community involvement in CHWs recruitment process like screening of candidates		
		1.1.4 Policy documents mention personal attributes as part of selection criteria		
		1.1.5 Policy documents include female gender prioritisation as part of selection criteria		
	1.2 Government sponsorship for CHW training	1.2.1 Government funds full tuition fees for CHW training		
		1.2.2 Government sponsors book and stationery allowance for CHWs undergoing training		
		1.2.3 Government sponsors accommodation allowance for CHWs undergoing training		
		1.2.4 Government sponsors transport allowance for CHWs undergoing training		
		1.2.5 Government sponsors stipend for CHWs undergoing training		
	1.3 CHWs’ training program accreditation	1.3.1 Government policy requires that CHW training institutions should be accredited by a national accreditation board		
		1.3.2 Government policy requires that CHW training curriculum is approved and accredited by a national accreditation board		
		1.3.3 Government policy requires that CHW training providers should be accredited by a national accreditation board		
		1.3.4 Government policy includes CHWs post-training accreditation be carried out by national accreditation board		
		1.3.5 Policy provides guidance on CHWs post qualification academic and career progression pathways		
	1.4 CHWs’ training modalities	1.4.1 Government policy documents provide guidance on duration of CHW training		
		1.4.2 Theory-focused knowledge and classroom component be made mandatory in policy documents		
		1.4.3 Practice-focused skills and field-based component be made mandatory in policy documents		
		1.4.4 Additional in-service, ongoing, refresher or continuous training are described in policy documents		
		1.4.5 End of course competency-based evaluation and certification is described in policy documents		
2. CHWs’ roles and responsibilities (Service delivery)	2.1 CHWs’ contractual agreements	2.1.1 Government policy indicates the need for CHWs to sign formal contractual agreements stipulating CHWs’ working conditions		
		2.1.2 Government policy indicates the need for CHWs to sign formal contractual agreements stipulating CHWs’ job responsibilities.		
		2.1.3 Government policy indicates the need for CHWs to sign formal contractual agreements stipulating CHWs’ working rights.		
		2.1.4 Government policy indicates the need for CHWs to sign formal contractual agreements stipulating duration of employment		
		2.1.5 Government policy indicates the need for CHWs to sign formal contractual agreements stipulating remuneration terms		
	2.2 CHWs’ roles and responsibilities	2.2.1 Policy documents indicate the need for role agreement amongst CHWs, community and health system		
		2.2.2 Policy documents describe the linkage between CHW cadres and the formal health system		
		2.2.3 Policy documents clarify the interventions and services delivered by CHWs to community and health system		
		2.2.4 Mode of transport for CHWs to access clients is described in the policy documents		
		2.2.5 CHWs’ services population coverage ratios and areas is clearly defined in policy documents		
	2.3 CHWs’ services linkage to health systems	2.3.1 Policy documents describe CHWs as the first tier or point of contact between community and health system		
		2.3.2 Policy guides how CHWs refer clients for health services that they cannot provide		
		2.3.3 Policy documents guide CHWs use of standardised referral tool for clients to take to the facility		
		2.3.4 Policy documents describe the transport system is availed to get clients to the referral facility		
		2.3.5 Policy documents defines health facilities, counter-refer patients to CHWs for follow-up		
3. CHWs’ remuneration (Health financing)	3.1 Funding for CHWs’ incentives	3.1.1 Government fully funds CHW incentives		
		3.1.2 NGOs and donors fully fund CHW incentives through the government		
		3.1.3 Government, donors, NGOs, and community collaboratively fund CHW incentives		
		3.1.4 Policy documents describe standardised package of financial incentives for CHWs		
		3.1.5 Policy documents describe standardised package of non-financial incentives for CHWs		
	3.2 Standardised package of financial incentives	3.2.1 Policy documents guides how CHWs should be salaried		
		3.2.2 Government policy documents guide per diem and stipends (meals, incidental expenses, travel, hotel costs) payments		
		3.2.3 Policy documents describe how performance-based allowances for CHWs like bonus will be paid		
		3.2.4 Cash payments from income generating projects is guided by policy documents		
		3.2.5 Allowance from funds from user fee and cost recovery systems and commodity sales is guided by policy documents		
3. CHWs’ remuneration (Health financing)	3.3 Standardised package of non-financial incentives	3.3.1 Policy guides modalities for preferential access to healthcare, accommodation and access to loans by CHWs		
		3.3.2 Policy guides modalities for CHWs opportunity for professional development like education, certificates, promotion to high position		
		3.3.3 Policy documents guide formal social recognition by other health workers and community		
		3.3.4 Policy documents describe how work aids (bicycles, motorbikes, cell phones, flashlights) are provided to CHWs		
		3.3.5 Policy guides how work uniforms (T-shirts, name tags, badges, umbrella, boots, raincoats, bags, backpacks) will be provided to CHWs		
4. CHW supervision (Leadership and governance)	4.1 CHWs’ supervision structure	4.1.1 CHWs are employed by the government		
		4.1.2 The government takes part in CHWs supervision		
		4.1.3 CHWs supervisors’ roles are described in the policy documents		
		4.1.4 Policy documents prescribe appropriate CHW supervisors training to prepare them to supervise CHWs		
		4.1.5 Policy documents guides how CHW supervisors get paid for supervising CHWs		
	4.2 CHWs’ supervision mechanisms and modalities	4.2.1 Community health work is managed across all levels of the health system		
		4.2.2 Appropriate supervisor-to-supervisee ratio is articulated in the policy documents		
		4.2.3 Policy documents define frequency of supervision for both CHWs and supervisor		
		4.2.4 Policy documents provide guidance on supervisionchecklists or tools used		
		4.2.5 Use of supervision feedback information is mandated in policy documents		
	4.3 CHWs’ supportive supervision strategies	4.3.1 Policy documents prescribe service delivery observation at community and facility as a supportive supervision strategy		
		4.3.2 Policy documents prescribe coaching and mentorship as a supportive supervision strategy		
		4.3.3 Policy documents provide community feedback as part of supervision strategies		
		4.3.4 Policy documents provide guidance on how technical and administrative support will be offered to CHWs		
		4.3.5 Policy documents provide guidance on the use of performance evaluation and feedback for continued improvement		
	4.4 CHWs’ performance management and evaluation by supervisors	4.4.1 Policy documents prescribe responsiveness to needs of the community as an indicator of CHWs’ performance		
		4.4.2 Policy documents prescribe resourcefulness as an indicator of CHWs’ performance		
		4.4.3 Policy documents prescribe teamwork and cooperativeness as an indicator of CHWs’ performance		
		4.4.4 Policy documents prescribe professional deportment as an indicator of CHWs’ performance		
		4.4.5 Policy documents prescribe service delivery as an indicator of CHWs’ performance		
5. CHWs’ information management (Health information)	5.1 CHWs’ data collection mechanisms	5.1.1 Data confidentiality and security articulated in government policy documents		
		5.1.2 Policy guides CHWs use digital technologies to enhance efficiency in data collection and processing		
		5.1.3 Policy documents stress the need for community and facility data consolidation		
		5.1.4 Government policy articulates how CHW generated data should be integrated into the national health information and management system		
		5.1.5 Policy documents provide guidance on how community and health system use data for problem-solving locally		
	5.2 CHWs’ role in data collection	5.2.1 Policy documents include routine collection of data as part of CHWs’ roles and responsibilities		
		5.2.2 Policy guides submission of community generated data to facility level		
		5.2.3 Policy documents prescribe use of standardised data collection tools		
		5.2.4 Policy guides use of collected data by CHWs to give feedback to community members		
		5.2.5 Policy guides use data by CHWs in problem-solving at community level		
	5.3 CHWs’ supervisor role in data collection	5.3.1 Policy documents include routine collection of data as part of CHWs’ roles and responsibilities		
		5.3.2 Policy guides training of CHW supervisors to verify and audit data for quality		
		5.3.3 Policy documents prescribe that CHW supervisors reports CHW generated data to next level of the health system		
		5.3.4 Policy guides use of data by CHW supervisors to provide feedback on CHWs’ performance		
		5.3.5 Policy guides use of data by CHW supervisors to inform programmatic improvement		
6. CHWs’ equipment and supplies (Medical products and technologies)	6.1 CHWs’ equipment and supply mechanisms	6.1.1 Policy guides how necessary equipment, supplies and job aids are availed to CHWs		
		6.1.2 Policy provides guidance on how CHWs conduct supplies inventory through manual or digital systems		
		6.1.3 CHWs’ commodities are included in the national supply chain plan as per policy guidelines		
		6.1.4 Mechanisms to replenish and replace CHW equipment and supplies are provided in policy documents		
		6.1.5 Policy guides supply forecasting using reliable data and standard tools for use by CHWs		
	6.2 CHWs’ equipment and supplies procedures	6.2.1 Policy documents provide adequate storage of commodities		
		6.2.2 Policy documents provide procedure for CHWs to access supplies		
		6.2.3 Policy documents provide procedures for stock-out management		
		6.2.4 Policy documents stress the need for regular CHWs’ supply updates for quality check		
		6.2.5 Policy documents guide procedures for accessing emergency back-up supplies		
6. CHWs’ equipment and supplies (Medical products and technologies)	6.3 CHWs’ equipment and supplies processes	6.3.1 CHWs obtain supplies using official request procedure as per policy guidelines		
		6.3.2 CHWs register all supplies and equipment obtained as per policy guidelines		
		6.3.3 Policy documents guide use of standard tools to order for resupplies		
		6.3.4 CHWs furnished with list of commodities for selected interventions that CHWs offer according to country’s policy guidelines		
		6.3.5 Policy guidance on safely disposal of medical waste generated through CHW service		

CHWs, community health workers.
